# Tailoring Multiple Strengthening Phases to Achieve Superior High-Temperature Strength in Cast Mg-RE-Ag Alloys

**DOI:** 10.3390/ma17040901

**Published:** 2024-02-15

**Authors:** Sicong Zhao, Erjun Guo, Kun Liu, Jingfang Li, Jianhua Liu, Mingyang Li

**Affiliations:** 1 Key Laboratory of Advanced Manufacturing and Intelligent Technology (Ministry of Education), School of Material Science and Chemical Engineering, Harbin University of Science and Technology, Harbin 150080, China; zscwr@163.com (S.Z.); erjunguo@126.com (E.G.); 2 Key Laboratory of Functional Inorganic Material Chemistry (Ministry of Education), School of Chemistry and Materials Science, Heilongjiang University, Harbin 150080, China; 3 Heilongjiang Beidacang Group Co., Ltd., Qiqihar 161000, China; bdcljh@126.com; 4 Qiqihar Heilong International Ice and Snow Equipment Co., Ltd., Qiqihar 161000, China; hrbust_lmy1987@126.com

**Keywords:** Mg alloys, precipitates, high-temperature performance, strengthening mechanism

## Abstract

Mg alloys with excellent high-temperature mechanical properties are urgently desired to meet the design requirements of new-generation aircraft. Herein, novel cast Mg-10Gd-2Y-0.4Zn-0.2Ca-0.5Zr-xAg alloys were designed and prepared according to the advantages of multi-component alloying. The SEM and XRD results revealed that the as-cast microstructures contained *α*-Mg grains, *β*, and Zr-containing phase. As Ag rose from 0 wt.% to 2.0 wt.%, the grain size was refined from 40.7 μm to 33.5 μm, and the *β* phase significantly increased. The TEM observations revealed that the nano-scaled *γ*′ phase could be induced to precipitate in the *α*-Mg matrix by the addition of Ag. The stacking sequence of lamellar *γ*′ phases is ABCA. The multiple strengthening phases, including *β* phase, *γ*′ phases, and Zr-containing particles, were effectively tailored through alloying and synergistically enhanced the mechanical properties. The ultimate tensile strength increased from 154.0 ± 3.5 MPa to 231.0 ± 4.0 MPa at 548 K when Ag was added from 0 to 2.0 wt.%. Compared to the Ag-free alloy, the as-cast alloy containing 2.0 wt.% Ag exhibited a minor reduction in ultimate tensile strength (7.0 ± 4.0 MPa) from 498 K to 548 K. The excellent high-temperature performance of the newly developed Mg-RE-Ag alloy has great value in promoting the use of Mg alloys in aviation industries.

## 1. Introduction

Materials science has become an important part of modern science and technology, and the development of novel materials has provided essential support for the development of various fields [1,2,3,4,5]. In particular, Mg alloys have attracted considerable interest in new energy vehicles, medical and rehabilitation equipment, digital equipment, and other fields. In recent years, Mg-RE series alloys have been extensively applied in aviation industries [6,7,8,9]. The use of lightweight magnesium alloy structural components has a remarkable effect on improving the maneuverability and fuel economy of various aircraft. Consequently, Mg-RE alloys are gradually replacing Al alloys, steel, and iron materials in numerous aviation structural components. Although Mg-light RE alloys, including Mg-Nd and Mg-Sm alloys, are considered highly competitive aviation engineering materials, their high-temperature applications are still limited. The previous research indicated that the heavy RE elements, including Gd and Y, are effective in improving the high-temperature performance of Mg alloys [10,11,12,13,14]. The maximum solubility level of Gd is 23.5 wt.% in the *α*-Mg matrix. The strength could increase linearly as the content of the Gd element increases. However, it is not always beneficial due to the increase in density and the decrease in ductility of Mg alloys. To address these deficiencies, the Gd element could be partially replaced with the Y, Zn, and Ca elements [15,16,17,18]. For cast Mg alloys, the most effective strengthening methods are second-phase strengthening and fine-grain strengthening. The second-phase strengthening is mainly through the addition of alloying elements to promote the formation of the second phase in the Mg alloy, and the obtained second phase has a higher hardness than the α-Mg matrix. Fine grain strengthening is mainly through the formation of a large amount of surface area of grain boundaries, and finer grains also improve the ability of the microstructure to coordinate deformation, which can increase the strength while improving the plasticity of the alloy. In addition, solid solution strengthening can also enhance the strength of the alloy, but its effect is not outstanding compared with the second-phase strengthening and fine-grain strengthening. In general, the three factors, including the second phase, more grain boundaries surface, or lattice distortion caused by solute atoms, essentially improve the strength of cast Mg alloys by hindering dislocation motion and suppressing twinning. Based on the above strengthening theory, a new cast Mg alloy will be rationally designed. According to the advantages of multi-component alloying, the RE, Zn, Ca, Zr, and Ag elements have favorable co-strengthening effects on Mg alloys. The RE element could accelerate strengthening phase formation and hinder the non-basal slip of Mg alloys [19,20,21]. Adding the Zn element could significantly reduce the stacking fault energy and improve the strength [22,23]. The minor content of the Ca element could further enhance the high-temperature resistance and flame resistance properties of Mg-RE alloys [24,25]. The Zr element is also the best choice to refine the microstructures [26,27]. Recently, some reports confirmed that Ag addition could significantly improve the strength of Mg alloys [28,29,30]. Especially for the solution-treated and aging-treated Mg alloys, the Ag could enhance their creep resistance and strength. Therefore, designing novel Mg-heavy RE-Ag alloys has important application value to satisfy the requirement of new aircraft. To our knowledge, the research of complex Mg-RE-Ag alloying systems has not been investigated sufficiently. The composition of the Mg-heavy RE-Ag alloys is usually composed of no more than quaternary components. Mg alloys with more components could obtain higher mechanical properties. However, the role of the Ag element in a complex Mg alloy system differs from that of binary or ternary Mg alloys. The interaction of Ag with RE, Zn, Ca, and Zr elements is not well studied in Mg alloys. Tailoring multiple strengthening phases in cast Mg-heavy RE alloys by Ag, RE, Zn, Ca, and Zr element alloying requires in-depth investigation. Moreover, many high-performance aero-engine components have complex structures. These components have small wall thickness and high dimensional tolerance requirements, and most of them will not be strengthened by further thermomechanical processing.

The main research aim of this work is to achieve cast Mg alloys with excellent mechanical properties by designing a novel Mg-RE-Ag alloy to meet the demand for lightweight alloys in the fields of aviation and aerospace. The mechanism of tailoring multiple strengthening phases to achieve superior high-temperature mechanical properties in the novel Mg-RE-Ag alloy was systematically investigated.

## 2. Materials and Methods

The abbreviation and compositions of Mg-10Gd-2Y-0.4Zn-0.2Ca-0.5Zr-xAg (x = 0, 0.5, 1.0, 1.5 and 2.0) alloys are listed in Table 1, which were measured by inductively coupled plasma-optical emission spectrometry (ICP-OES, Thermo iCAP 7400, Thermo Fisher Scientific Inc., Hillsboro, OR, USA). The raw materials included pure Mg (>99.98%), Ag (>99.99%), Zn (>99.99%), and Mg-30 wt.%Gd, Mg-30 wt.%Y, Mg-30 wt.%Zr, and Mg-30 wt.%Ca alloys, and the source of these materials is Yueyang Yuhua Metallurgical New Materials Co. Ltd. in China. The alloys were melted in an electric resistance furnace under a mixed atmosphere of CO_2_ and SF_6_ with a ratio of 100:1. The pure Mg was completely melted at 993 K. Then the temperature increased to 1023 K, while all alloy materials except the Mg-30 wt.%Zr alloy were added to the crucible. The temperature rose to 1053 K after adding the Mg-30 wt.%Zr alloy and stirring the melt for 5 min. Finally, the melt was poured into a steel mold when the temperature dropped to 993 K. All raw materials, crucibles, and molds were preheated to 473 K. The scanning electron microscope (SEM, Apreo C, Thermo Fisher Scientific Inc., Hillsboro, OR, USA) was used to observe the microstructure of the alloys, and the operating voltage and beam current are 20 kV and 0.8 nA, respectively. The SEM observation included secondary electron (SE) and backscattered electron (BSE) modes. The energy dispersive spectroscopy (EDS, Oxford Instruments, London, UK) was utilized to analyze the distribution and content of alloying elements in microstructure. The transmission electron microscope (TEM, JEM-2100, JEOL Co. Ltd., Tokyo, Japan) was employed to observe and analyze the microstructure morphology and its diffraction patterns, especially to determine the type of nano-scaled phases. The operating voltage and beam current of TEM are 200 kV and 101 μA, respectively. The TEM specimens were mechanically ground to 50 μm and then subjected to ion thinning under the voltage and angle of 3.5 kV and 3°, respectively. The X-ray diffraction (XRD, X’Pert PRO, PANalytical B.V., Almelo, The Netherlands) was equipped with Cu Ka at 40 kV to analyze the constitutive phases in the alloys. The MDI Jade software (Materials Data Inc., Livermore, CA, USA) analyzed the XRD results with the pdf database (2004 version). The proportion of constitutive phases based on quantitative metallographic calculations was used as a preliminary reference. The result of quantitative metallography is the percentage of the area occupied by the constitutive phases in the metallographic images. In order to ensure the reliability of the results, the Rietveld method is further used to determine the proportion of constitutive phases based on the obtained diffractograms. The Rietveld method enables the calculation of the mass percentage of the constitutive phase in the alloys. The average grain size was determined using the standard lineal intercept test according to ASTM-E112. In order to minimize measurement errors, no less than five low-magnification images were used to calculate the average grain size. The given size is the average diameter of grains. Brinell hardness (HB, HB3000, Sinotest Equipment Co., Ltd., Changchun, China) test was performed under 225 N for 15 s. The tensile test (MTS E44.304, MTS Systems Co., Eden Prairie, MN, USA) was performed to measure the tensile properties at room temperature (298 K), 498 K, 523 K, and 548 K, respectively. The gauge width, thickness, and length of tensile samples are 2 mm, 2 mm, and 6 mm, respectively.

## 3. Results and Discussion

### 3.1. Microstructures

**Figure 1:**
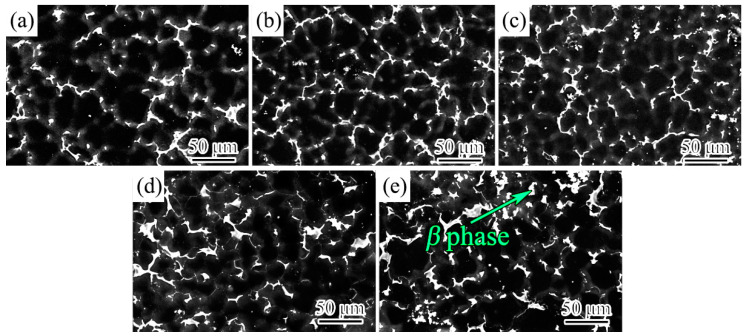
SEM morphology of (**a**) 0 Ag alloy, (**b**) 0.5 Ag alloy, (**c**) 1.0 Ag alloy, (**d**) 1.5 Ag alloy, and (**e**) 2.0 Ag alloy.

**Figure 2:**
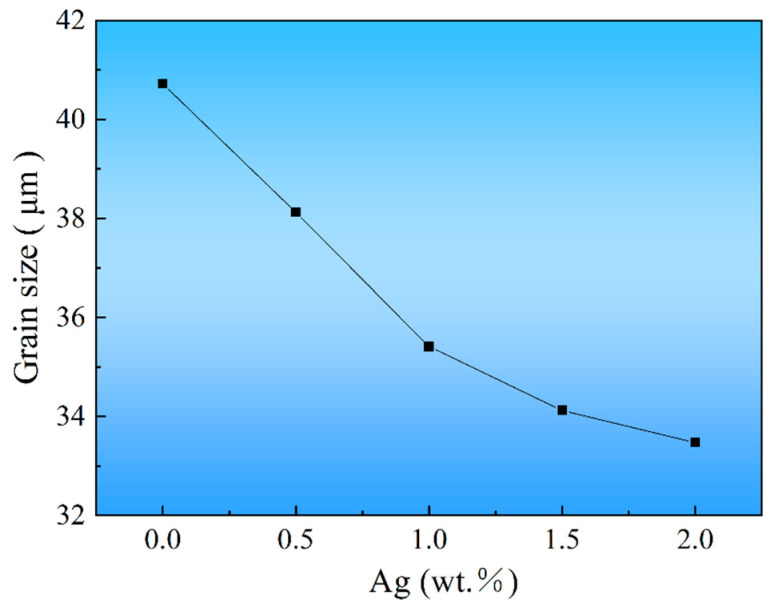
Grain size of the alloys varies with Ag content.

Figure 1 shows the SEM morphology of as-cast Mg-10Gd-2Y-0.4Zn-0.2Ca-0.5Zr-xAg alloys under SE mode. The microstructures of the alloys are composed of *α*-Mg grains and network phase on the grain boundaries. Based on the chemical composition of the alloys, the morphology of the network phase, and in combination with the previous report [6], it can be preliminarily determined that the network phase on the grain boundary may be the *β* phase. Subsequently, the network phase will be further confirmed to be the β phase by XRD, EDS, TEM, and SAED analyses. The Ag-free alloy exhibited extremely coarse grains (40.7 μm). The grain gradually refined as the amount of Ag element increased. As seen in Figure 2, the average grain size was determined using the standard lineal intercept test. The statistical results of grain size indicated that the grain refinement effect was most pronounced when the addition of Ag was within 1.0 wt.%. After Ag increased to 2.0 wt.%, the grains could still be further refined, but the refining effect gradually weakened. The finest grain size of 33.5 μm was achieved when the Ag was increased to 2.0 wt.%. The key reason for grain refinement could be attributed to the fact that the growth restriction factor (*Q* value) of the as-cast alloys can be improved by the addition of Ag. Generally, the high *Q* value corresponds to a high establishment rate in the constitutional supercooling region, resulting in finer grain [31].

**Figure 3:**
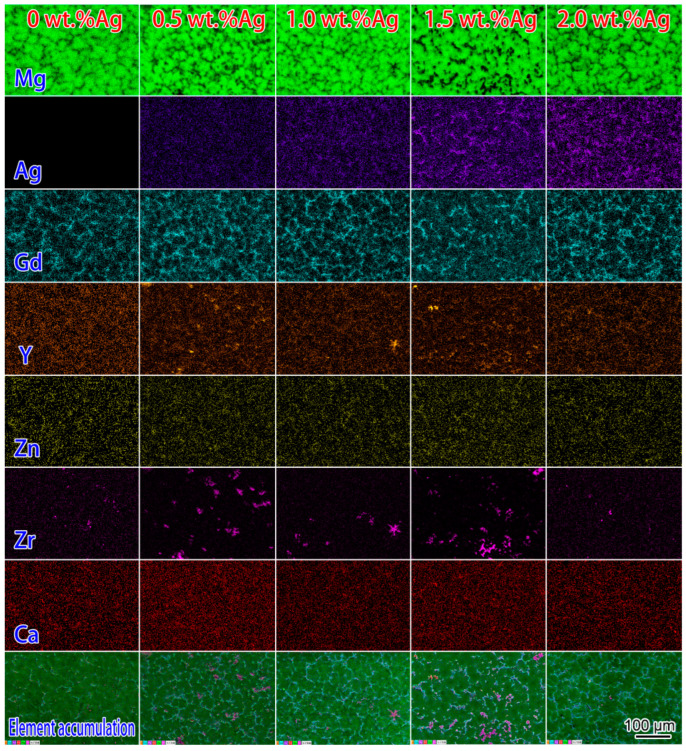
Elemental distribution of as-cast alloys with different contents of Ag element.

The EDS mapping was employed to measure the Ag, Gd, Y, Zn, Ca, and Zr element distribution. Figure 3 demonstrated that Ag mainly segregated at grain boundaries and formed the irregular network phase. Moreover, the contrast of Ag mapping in the network phase also increased with the addition of Ag. It was also proved that Ag promoted the formation of the network phase. The distribution of the Gd element was similar to that of Ag. Obviously, Gd and Ag elements formed the irregular network phase together. The Y element was mainly segregated at the grain boundary, but the distribution of the Y element was not uniform. The Y element is more likely to form unevenly distributed rare earth oxides than the Gd element during the melting and casting process. Most of the Zr elements are segregated in *α*-Mg grain interior. Additionally, trace amounts of Gd, Y, Zn, Ag, and Ca elements are contained in the *α*-Mg matrix.

**Figure 4:**
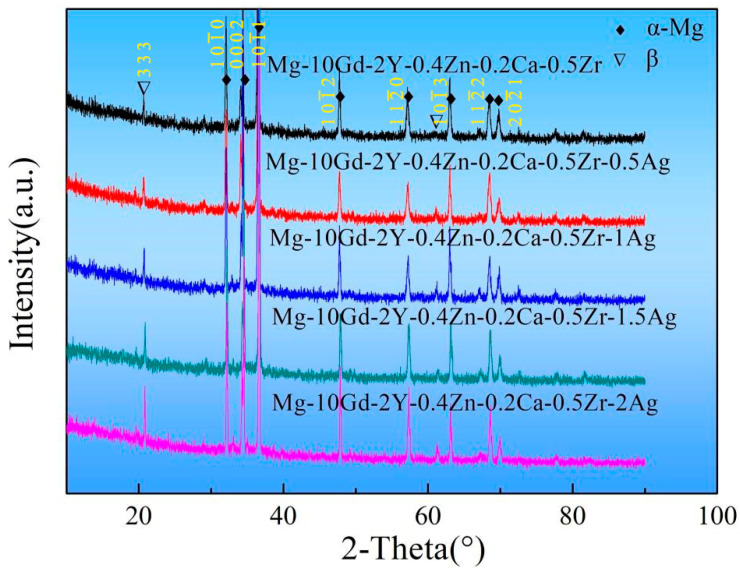
XRD results of as-cast Mg-10Gd-2Y-0.4Zn-0.2Ca-0.5Zr-xAg alloys.

**Figure 5:**
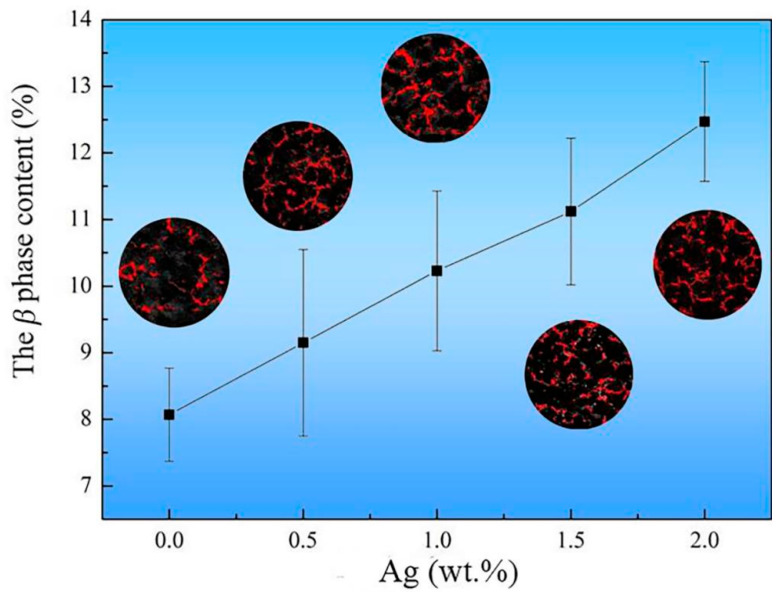
The *β* phase in the as-cast alloys as a function of Ag content.

The XRD results (Figure 4) revealed that the irregular network intermetallic compound was *β* phase. As the Ag content increased, there was a noticeable increase in the peak value of the *β* phase. The statistical content of the *β* phase is given in Figure 5. The results are based on the method of quantitative metallography, and the values being counted are the percentage of the area in the metallographic images occupied by the *β* phase. The area proportion of the *β* phase was only 8.07% in the Ag-free alloy. The area proportion of the *β* phase can be up to 12.47% when the Ag increases to 2.0 wt.%. In addition, we also calculated the mass fraction percentage of *β* phase in the alloy using the Rietveld method. The same statistical trends were obtained, and the mass fraction percentage of *β* phase in the alloy increased from 2.9 wt.% to 6.3 wt.% when the addition of Ag element was increased from 0 wt.% to 2 wt.%. Accordingly, all the above results indicated that Ag is an effective element in promoting the formation of *β* phase.

**Figure 6:**
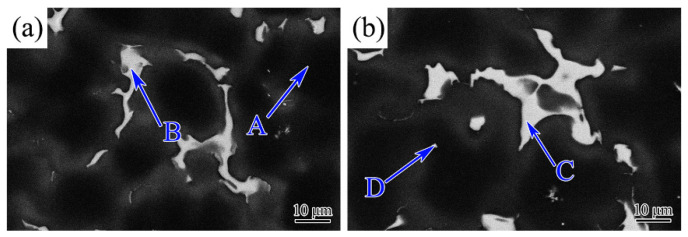
The SEM morphology of as-cast (**a**) 0 Ag alloy and (**b**) 2.0 Ag alloy. The points of A, B, C and D are the positions for EDS analysis.

Figure 6 shows typical SEM micrographs of Ag-free alloy and 2.0 Ag alloy under the BSE mode. The EDS results of the selected positions in Figure 6 are given in Table 2. The EDS is an effective method for analyzing the composition of the second phase in microstructures. Although EDS analysis is only semi-quantitative, its measurement accuracy can meet the study of elemental segregation and assist in determining the composition of the constitutive phase. Point A proved that a small amount of Gd, Y, Zn, Zr, and Ca elements existed in the *α*-Mg matrix of the Ag-free alloy. The major elements of Point B were Mg and Gd, as well as a small amount of Zn, Y, and Ca elements. The atom ratio of Mg and alloying elements (Gd, Y, Zn, and Ca) approached 5:1. The EDS analysis determined that the *β* phase in the Ag-free alloy was Mg_5_ (Gd, Y, Zn, Ca). As for the 2.0 Ag alloy, Point C confirmed that the composition of the *β* phase contained the Ag element except for Mg, Gd, Y, Zn, and Ca elements. The atom ratio of Mg and alloying elements (Gd, Ag, Y, Zn, Ca) was also approached 5:1 in the *β* phase of 2.0 Ag alloy. Consequently, the chemical composition of the *β* phase changed to Mg_5_ (Gd, Ag, Y, Zn, Ca) after the Ag addition. Moreover, point D showed that some particles rich in the Zr element appeared in the *α*-Mg matrix. The Zr-containing phase was helpful in refining the grain.

**Figure 7:**
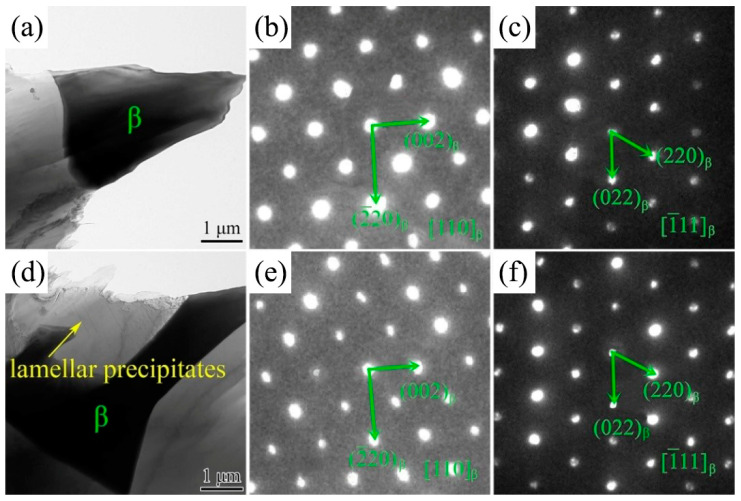
TEM micrographs and corresponding SAED patterns of the *β* phase. (**b**,**c**) are the SAED patterns of (**a**); (**e**,**f**) are the SAED patterns of (**d**).

TEM studies were carried out to analyze the morphologies and structures of the *β* phase in 2.0 Ag alloy. The typical *β* phase morphologies were observed, as shown in Figure 7a,d. The SAED patterns were obtained under the electron beam paralleling the (110)*_β_* direction and ($\overset{-}{1}$11)*_β_* direction. According to diffraction results, the *β* phase was determined to be fcc structure. Calculation with SAED patterns revealed that lattice parameters of the *β* phase were a*_β_* = 2.23 nm. The results were consistent with previous studies on Mg-Gd series alloys [32,33]. Therefore, the TEM characterization demonstrated that the irregular network phase crystal structure was not changed after the addition of Ag.

**Figure 8:**
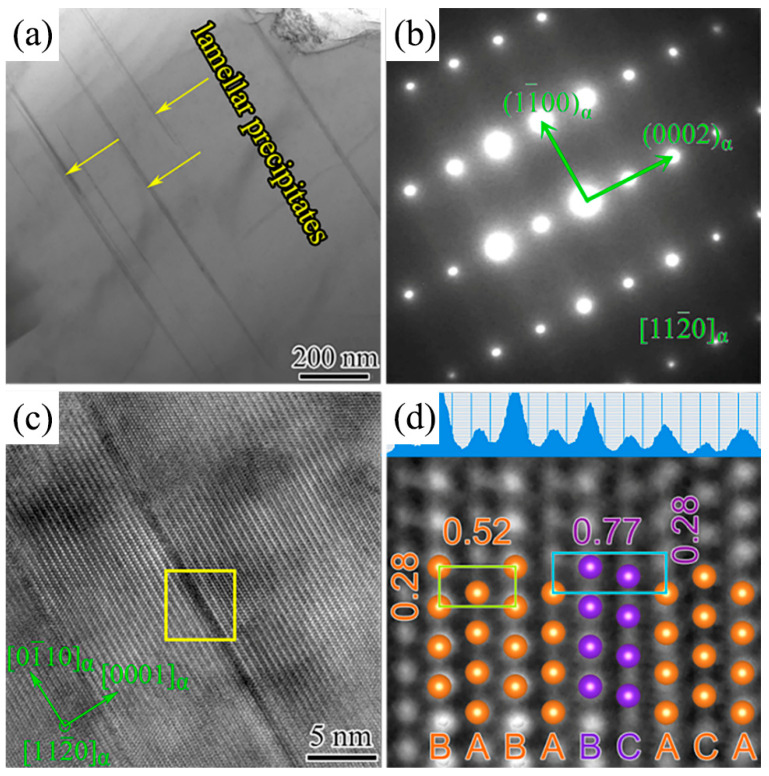
TEM images of as-cast 2.0 Ag alloy along (11$\overset{-}{2}$0)*_α_* direction. (**a**) High-magnification TEM image; (**b**) SAED patterns of the (**a**); (**c**) HRTEM image; (**d**) IFFT image of the selected area from (**c**).

Furthermore, Figure 7d presents numerous nano-scaled lamellar precipitates. These precipitates were no less than 1 μm in length, but their thickness is extremely small and difficult to distinguish at low-magnification TEM images. Figure 8a shows high-magnification TEM micrographs of the lamellar precipitates viewed along (11$\overset{-}{2}$0)*_α_* zone axis. Figure 8b shows the corresponding SAED patterns of Figure 8a. The lamellar precipitates formed on the basal plane of the *α*-Mg matrix with an extremely large aspect ratio based on the TEM and SAED analysis of the as-cast 2.0 Ag alloy. In Figure 8c, the lamellar precipitates had only four basal atomic layers in thickness. Figure 8d shows an inverse Fourier-filtered transformation (IFFT) image. Notably, the atomic stacking sequence of lamellar precipitates can be clearly identified as ABCA. According to this special stacking sequence and habit plane of the lamellar precipitates, it was proposed that the lamellar precipitates can be identified as *γ*′ phase. The *γ*′ phase was commonly formed in the aged Mg-RE-Zn alloys and rarely observed in the as-cast alloys. Adding Ag could induce the formation of the *γ*′ phase. The quantitative measurement revealed that the thickness of the *γ*′ phase was 0.77 nm in as-cast 2.0 Ag alloy. The thickness of the *γ*′ phase in aged Mg-RE-Zn alloys was 0.78 nm, which had a similar thickness to that of ABAB atomic layers in the *α*-Mg matrix. The stacking fault distortion of the *γ*′ phase achieved in this work could result in a stronger strength response.

### 3.2. Mechanical Properties

#### 3.2.1. Hardness

**Figure 9:**
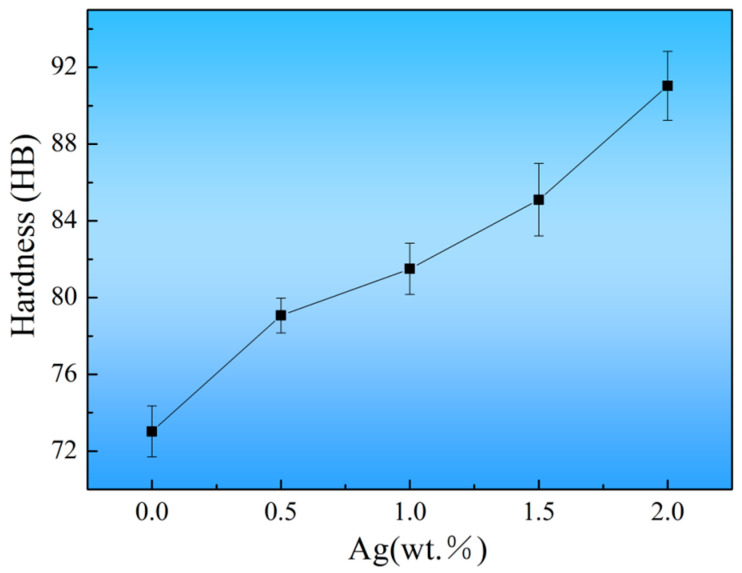
The hardness of the as-cast alloys as a function of Ag content.

Figure 9 indicated that the hardness of as-cast alloys increased from 73.0 ± 3.0 HB to 91.0 ± 4.5 HB with the addition of Ag from 0 to 2.0 wt.%. The following factors are primarily responsible for the increased hardness. First, the Ag was critical in fine-grain strengthening. The refined grain increased the area of grain boundaries, which act as strong barriers to slip transmission, hence contributing to a higher hardness. Second, the Ag addition could significantly promote the formation of the strengthening phase. The *β* phase and *γ*′ phase could effectively pin dislocation and restrict sliding [34]. Hence, the second phase strengthening was also an essential way to enhance the hardness of as-cast alloys. Additionally, the Zr-containing phase and a small amount of the solute atom existing in the *α*-Mg matrix also have an effect on increasing the hardness of the alloys.

#### 3.2.2. Tensile Properties

**Figure 10:**
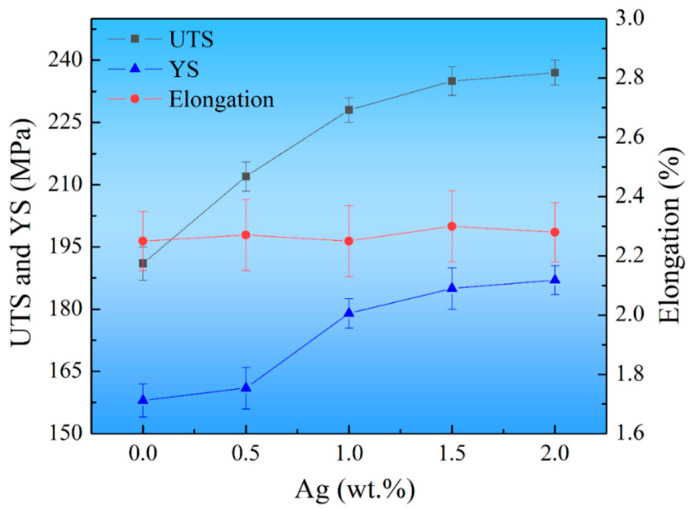
Room-temperature tensile properties of the as-cast alloys as a function of the Ag content.

Figure 10 shows the room-temperature tensile properties of the as-cast alloys. The ultimate tensile strength (UTS) increased from 191.0 ± 4.0 MPa to 237.0 ± 3.0 MPa, the yield strength (YS) increased from 158.0 ± 4.0 MPa to 187.0 ± 3.5 MPa, and the elongation was in the range of 2.1% to 2.4% by adding Ag from 0 to 2.0 wt.%. The impact trend of Ag on the YS of the alloy is generally consistent with the trend of the UTS. Obviously, adding Ag can significantly enhance the room-temperature strength. Meanwhile, the elongation did not change noticeably. Moreover, the room-temperature strength of the as-cast Mg-heavy RE-Ag alloys achieved in this study is significantly higher than most reported as-cast Mg alloys [35,36,37,38,39,40,41,42,43,44]. Like the increase in hardness, the tensile strengthening at room temperature could also be caused by the grain refinement and the increased multiple strengthening phases.

**Figure 11:**
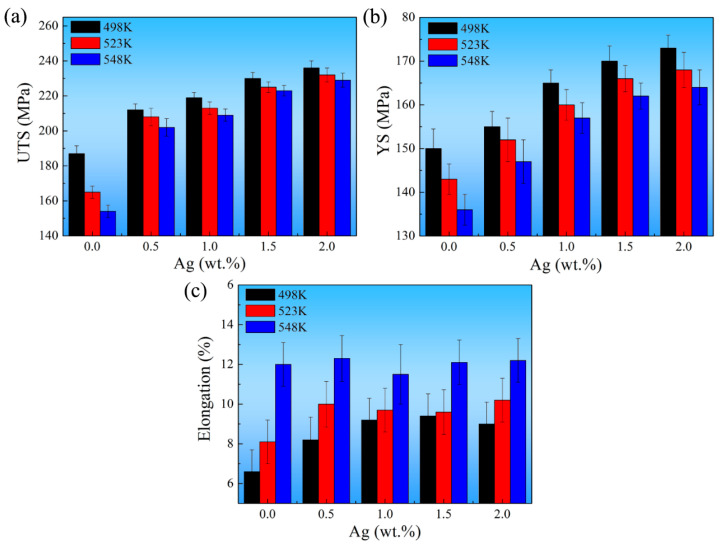
High-temperature tensile properties of the as-cast alloys: (**a**) UTS; (**b**) YS; (**c**) Elongation.

The tensile properties of the as-cast alloys at high temperatures are provided in Figure 11. It can be known that the UTS and YS decreased at high temperatures in comparison with that at room temperature (Figure 10), and the elongation increased gradually when the tensile temperature increased from room temperature to 548 K. Notably, the UTS of the Ag-free alloy fell dramatically from 187.0 ± 4.5 MPa to 154.0 ± 3.5 MPa when the temperature increased from 498 K to 548 K, and the drop was 33 MPa. However, the UTS of the 2.0 Ag alloy reduced from 236.0 ± 4.0 MPa to 229.0 ± 4.0 MPa, which only decreased by 7.0 MPa. Therefore, without a doubt, adding Ag to Mg-heavy RE alloys will greatly enhance their mechanical properties at high temperatures. It could be caused by grain refinement and the increase of the multiple strengthing phases. For the previously reported heat-treated Mg-RE alloys [45,46,47,48], their strengthening phases are mainly metastable *β*″, *β*′, and *β*_1_ series precipitates, which are less stable at high temperatures than the *β* phase. Compared to as-cast Mg-RE alloys, the high-temperature strength of these heat-treated alloys is more likely to decline in long-term service at high-temperature conditions. This is also an essential advantage of lightweight as-cast Mg-RE-Ag alloys for long-term applications in high-temperature environments. Moreover, the cast alloy has a higher content of *β* phase than the heat-treated alloy on the grain boundary. Grain boundary slip may be effectively inhibited by a higher *β* phase content. Additionally, the formation of *γ*′ phase and Zr-containing phase can also impede dislocation slip within *α*-Mg grains effectively. The above reasons are the key to the superior mechanical properties of Mg-RE-Ag alloys compared to conventional Mg-RE alloys. Consequently, the *β* phase, *γ*′ phase, and Zr-containing phase significantly inhibited grain boundary slip and dislocation slip at high temperatures, leading to increased high-temperature strength. Figure 11c shows that the elongation increased considerably when the temperature went up from 498 K to 548 K. Non-basal slip could be effectively activated during the high-temperature tensile process. As a result, the number of slip systems increased significantly above room temperature. Meanwhile, the critical resolved shear stress was reduced as temperature increased, accelerating the coordinating deformation of the *α*-Mg matrix [49,50,51].

### 3.3. Fracture Morphologies

**Figure 12:**
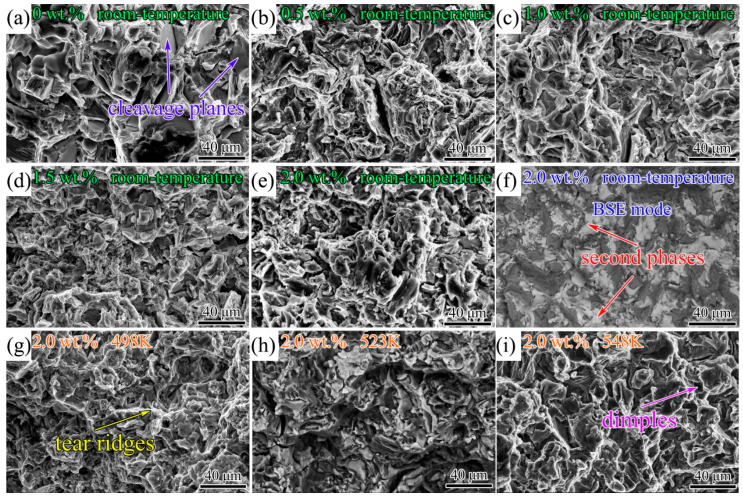
SEM micrographs of tensile fractures; (**a**) 0 Ag alloy, (**b**) 0.5 Ag alloy, (**c**) 1.0 Ag alloy, (**d**) 1.5 Ag alloy and (**e**) 2.0 Ag alloy tensiling at room temperature observed in SE mode; (**f**) observed in BSE mode corresponding to the figure (**e**); 2.0 Ag alloy tensiling at high temperature: (**g**) 498 K, (**h**) 523 K and (**i**) 548 K observed in SE mode.

Figure 12 presents the fracture surfaces of the as-cast alloys. Figure 12a–e show the second electron images of the room-temperature tensile fractured surfaces. The fractured surfaces mainly consisted of cleavage planes, which could be classified as brittle fractures. According to the backscattering electron mode (Figure 12f) corresponding to Figure 12e, it was clearly seen that the highlight zone in the BSE mode was covered by the *β* phase, which could be the source of cracks. Figure 12g–i show the SE images of the high-temperature tensile fractured surfaces for the as-cast 2.0 Ag alloys. It can be observed that a few tear ridges and some dimples formed in the fracture surface due to the softening effect of the *α*-Mg matrix at high temperatures. The addition of Ag reduced the size of the cleavage planes and, therefore, refined the fracture microstructure.

## 4. Conclusions

The multiple strengthening phases, including *β* phase, *γ*′ phases, and Zr-containing particles, synergistically enhanced the mechanical properties of the 10Gd-2Y-0.4Zn-0.2Ca-0.5Zr-xAg alloys. The addition of Ag can significantly promote the formation of the *β* phase. The composition of the *β* phase is Mg_5_(Gd, Ag, Y, Zn, Ca). The formation of *γ*′ phase on the basal plane of the *α*-Mg matrix was induced by the addition of Ag, and the stacking sequence of *γ*′ phase is ABCA. The grain of as-cast alloys was gradually refined from 40.7 μm to 33.5 μm by adding Ag from 0 to 2.0 wt.%. The addition of Ag reduced the size of the cleavage planes and, therefore, refined the fracture microstructure. The fractured surfaces mainly consisted of cleavage planes, which could be classified as brittle fractures. The 2.0 Ag alloy achieved the highest UTS of 231.0 ± 4.0 MPa at 548 K and exhibited a slight decrease in UTS of 7.0 ± 4.0 MPa from 498 K to 548 K with respect to Ag-free alloy. The novel Mg-RE-Ag alloy is of great significance in reducing structural weight and improving the performance of aero-engine components.

## Figures and Tables

**Table 1:** The abbreviation and compositions of the alloys.

Alloy	Ag		Gd		Y		Zn		Zr		Ca		Mg
	wt.%	at.%	wt.%	at.%	wt.%	at.%	wt.%	at.%	wt.%	at.%	wt.%	at.%	—
0 Ag	—	—	9.89	1.70	1.59	0.48	0.41	0.17	0.51	0.15	0.15	0.10	Bal.
0.5 Ag	0.52	0.13	10.05	1.74	1.61	0.49	0.39	0.16	0.52	0.16	0.16	0.11	Bal.
1.0 Ag	1.04	0.26	9.88	1.72	1.56	0.48	0.40	0.17	0.51	0.15	0.15	0.10	Bal.
1.5 Ag	1.50	0.38	10.11	1.77	1.60	0.49	0.41	0.17	0.50	0.15	0.15	0.10	Bal.
2.0 Ag	2.05	0.53	10.25	1.80	1.59	0.49	0.41	0.17	0.51	0.15	0.16	0.11	Bal.

**Table 2:** EDS analysis (at. %) of the typical positions in Figure 6.

Point	Mg		Ag		Gd		Y		Zr		Zn		Ca	
	at.%	wt.%	at.%	wt.%	at.%	wt.%	at.%	wt.%	at.%	wt.%	at.%	wt.%	at.%	wt.%
A	97.67	88.44	—	—	1.62	9.49	0.37	1.23	0.12	0.41	0.11	0.27	0.11	0.16
B	84.31	50.11	—	—	10.78	41.46	1.95	4.24	—	—	2.08	3.33	0.88	0.86
C	83.65	49.35	2.41	6.31	9.39	35.84	2.44	5.27	0.16	0.35	1.61	2.56	0.34	0.33
D	83.91	57.60	0.24	0.73	0.52	2.31	—	—	15.20	39.16	0.08	0.15	0.05	0.06

## Data Availability

Data will be made available on request.
